# Exposure to Rivals Does Not Affect Male Mate Choice in *Drosophila melanogaster*

**DOI:** 10.1002/ece3.72284

**Published:** 2025-10-10

**Authors:** Harini Shankar, Avigayil Lev, Rachel S. Halpern, Thahiya Hassan, Mingxi Jiang, Isabella M. Martinez, Keane L. Robinson, Lauren E. Subramaniam, Rachel Zackai, Alison Pischedda

**Affiliations:** ^1^ Department of Biology, Barnard College Columbia University New York New York USA; ^2^ Columbia College Columbia University New York New York USA

**Keywords:** behavioral plasticity, *Drosophila melanogaster*, male mate choice, male–male competition, mating duration, sexual selection

## Abstract

Male–male competition can affect male mate choice in different ways, potentially leading to stronger preferences when males benefit from investing more resources in fewer but higher‐quality matings or weaker preferences if choosiness could lead to missed mating opportunities. As in many insects, *Drosophila melanogaster* males show both pre‐ and postcopulatory preferences for larger, higher‐fecundity females. Exposure to rivals alters some aspects of male courtship and mating behavior in this species, but it is unknown whether male mate choice is similarly affected by increased competition. We tested whether perceived competition influenced the strength of male precopulatory and postcopulatory preferences for large females by housing *D. melanogaster* males with or without rivals before they encountered females that differed in size. Males from both treatments exhibited equally strong courtship preferences for large females and extended matings to a similar degree with large versus small females, indicating that exposure to rivals did not affect these components of male mate choice. There was also no difference in offspring production between males from the two treatments. Our findings suggest that male preferences for large females are relatively stable across social environments in *D. melanogaster* , supporting the hypothesis that strong, consistent preferences for high‐quality mates can be maintained despite variation in competitive contexts.

## Graphical abstract

We tested whether perceived competition affected male mate choice for large, high‐fecundity females in *Drosophila melanogaster* . All males showed consistently strong preferences for large females, regardless of whether or not they had previously encountered rivals.

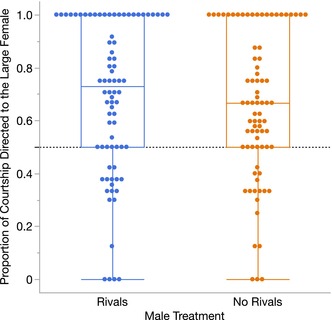

## 1 Introduction

Although sexual selection research has traditionally focused on female mate choice, male mate choice is now recognized as both prevalent and significant across a range of taxa (Edward and Chapman 2011). Males frequently exhibit preferences for female traits associated with fecundity or fertility, likely because choosing high‐quality mates can increase male reproductive success (Bonduriansky 2001; Edward and Chapman 2011; Pollo et al. 2022). These male preferences can be seen both before and after copulation. Precopulatory male mate choice occurs when males direct more courtship or mating effort toward particular females (Edward and Chapman 2011), while postcopulatory, or “cryptic”, male mate choice involves differential allocation of reproductive resources, such as sperm or seminal fluid, after mating has begun (Pitnick and Brown 2000; Reinhold et al. 2002; Edward and Chapman 2011). Because male mate choice can shape genetic diversity and influence the rate of adaptive evolution within populations (Long et al. 2009; Edward and Chapman 2011; Pollo et al. 2022), it is important to understand the factors that drive variation in male preferences.

One factor that is predicted to affect male mate choice is competition between males for access to females or fertilizations. This could either strengthen or weaken male preferences, depending on the ecological and social context (Fitzpatrick and Servedio 2018). First, when competition between males increases, males could be selected to invest more in mating effort (e.g., court with higher intensity, transfer larger ejaculates, etc.). If this increased investment reduces males' ability to mate with multiple females, males may become choosier to maximize their reproductive success from a limited number of matings (Bretman, Gage, and Chapman 2011; Edward and Chapman 2011; Fitzpatrick and Servedio 2018). Alternatively, if increased male competition causes choosy males to miss out on potential mating opportunities, this could reduce the net fitness benefits of mate choice, favoring weaker preferences in males (Fawcett and Johnstone 2003; Servedio and Lande 2006; Servedio 2007; Edward and Chapman 2011; Fitzpatrick and Servedio 2018; Pollo et al. 2022). There is empirical support for both hypotheses: increased competition strengthened male preferences for high‐fecundity females in fungus beetles (Formica et al. 2016) and orb‐weaving spiders (Bel‐Venner et al. 2007), but weakened male preferences in mosquitofish (Wong and McCarthy 2009; Mautz and Jennions 2011) and threespine sticklebacks (Candolin and Salesto 2009). These contrasting findings emphasize that the predicted relationship between male competition and male mate choice can change depending on the consequences of competition and the benefits that males receive from being choosy.

Male mate choice for larger females is common in ectothermic species (Bonduriansky 2001; Edward and Chapman 2011), where female body size and fecundity are often positively correlated (Honěk 1993; Roff 2002). This preference has been particularly well documented in insects (Bonduriansky 2001), including the fruit fly, *Drosophila melanogaster* . *D. melanogaster* males direct more courtship toward larger females than smaller females (Sinclair et al. 2021; Lev and Pischedda 2023) and typically mate for longer with larger females (Lefranc and Bundgaard 2000; Lüpold et al. 2011; Anastasio et al. 2023). These behaviors can provide both direct and indirect fitness benefits to males: compared to smaller females, larger females produce more offspring (Long et al. 2009; Lev and Pischedda 2023; Lev et al. 2025) and have daughters with higher reproductive success (Lev et al. 2025). Although there is genetic variation in the strength of male preferences for larger females in *D. melanogaster* (Freed et al. 2025), plasticity in this preference appears relatively weak, as it is unaffected by the male's condition (Lev and Pischedda 2023) or prior sexual experience (Sinclair et al. 2021; Anastasio et al. 2023). Given this lack of plasticity, it remains unclear whether male mate choice is affected by male competition in this species.

There is reason to expect that competition could influence male mate choice, since perceived competition is known to affect several male reproductive behaviors in *D. melanogaster* . After being exposed to potential rivals, males take longer to initiate courtship (Dore et al. 2021) and increase their mating duration (Bretman et al. 2009, 2010; Bretman, Westmancoat, et al. 2011; Bretman, Westmancoat, and Chapman 2013; Wigby et al. 2009; Rouse and Bretman 2016; Dore et al. 2021; Fowler et al. 2022) compared to males that have not been exposed to rivals. This latter result may reflect a plastic response to anticipated sperm competition, given that males exposed to rivals also transferred higher quantities of seminal fluid proteins during mating (Wigby et al. 2009; Hopkins et al. 2019). However, the adaptive benefits of these plastic responses to competition remain unclear, with some studies reporting increased male reproductive success after encountering rivals (Bretman et al. 2009, 2012; Bretman, Westmancoat, et al. 2011; Bretman, Westmancoat, Gage, and Chapman 2013; Hopkins et al. 2019), while others find no such effect (Nandy et al. 2016; Dore et al. 2020; Fowler et al. 2022). Nevertheless, the fact that perceived competition changes male courtship and mating behaviors suggests that it could similarly influence the strength of pre‐ and postcopulatory male preferences.

Here, we investigated whether male competition influences male mate choice for large females in *D. melanogaster* by altering the number of rivals that experimental males were exposed to before encountering females that differed in size. We then measured the strength of their pre‐ and postcopulatory preferences by comparing two main metrics of male choice: the proportion of courtship directed toward the large female versus the small female (our measure of precopulatory male mate choice) and the difference in mating durations with large versus small females (our proxy for postcopulatory male mate choice). Because the predicted impact of male competition depends on male fitness benefits with preferred females, we also compared the number of offspring sired by males after they encountered competitive or noncompetitive environments. If increased competition favors stronger male preferences in this system, males that have been exposed to rivals should increase courtship and/or mating duration with large females, while the opposite pattern would indicate that competition favors weaker male preferences in this system. Understanding how male competition influences male mate choice integrates two interrelated components of sexual selection, offering insight into the plasticity of this process and how it may shift under different evolutionary pressures.

## 2 Methods

### 2.1 *Drosophila* Population and Maintenance

For all experiments, we used *D. melanogaster* flies from the wild type, outbred LH_M_ population. A detailed description of this population and its maintenance protocol can be found in Rice et al. (2005). Briefly, this population is maintained on 2‐week, discrete lifecycles in 25 mm diameter vials with 5–10 mL of a cornmeal‐yeast‐molasses medium. On day 12 post‐egg deposition, adult flies from 56 vials are collected, pooled, anesthetized using CO_2_, and redistributed into 56 fresh vials in groups of 16 males and 16 females per vial (1,792 flies total). Each vial contains food medium with 6.4 mg of live yeast on the surface. After 2.5 days, the flies are transferred to fresh unyeasted vials for an 18‐h egg laying period, after which the adults are discarded. Excess eggs are then manually removed to ensure a standard density of 150–200 eggs per vial to begin the next generation. All experimental flies were held in an incubator at 25°C with 50%–70% humidity on a 12‐h light/12‐h dark cycle in vials containing food medium. All behavioral experiments were conducted within 2 h of the incubator lights turning on.

### 2.2 Creating Large and Small Females

To create large and small females for male mate choice trials, we manipulated larval density during the developmental period of the flies using the same approach as Byrne and Rice (2006). We first placed 16 male–female pairs of sexually mature LH_M_ flies into small egg collection cages (3.75 cm diameter by 5.8 cm height; Genesee Scientific) fitted with a 35 mm diameter Petri dish containing 5 mL of food medium, which the females oviposited on. After approximately 18 h, adult flies were discarded, and eggs were randomly collected from each Petri dish. To generate small‐bodied females, we transferred 100 eggs into vials containing 1 mL of food medium, and to generate large‐bodied females, we placed 50 eggs into vials with 10 mL of food. Because development proceeds more slowly under higher‐density conditions, eggs for the small female treatment were collected 1 day earlier than those for the large female treatment to synchronize eclosion timing.

Vials were incubated under standard conditions for 8 or 9 days (for large or small females, respectively), and females were collected using light CO_2_ anesthesia within 6 h of eclosion to ensure virginity. All females were then held in vials containing a small amount of food medium at a density of 10 per vial for 4 days prior to the experiment. Female virginity was confirmed by checking holding vials for larvae and discarding any vials in which larvae were present.

This method for altering adult body size consistently produces large and small females with non‐overlapping, visually distinguishable size distributions that fall within the range of naturally occurring body sizes in the LH_M_ population (Anastasio et al. 2023; Stewart et al. 2024). Males readily court and mate with females from both size classes, and male mate choice preferences are consistent in direction and magnitude whether female size is naturally sampled or experimentally induced (Long et al. 2009; Sinclair et al. 2021; Anastasio et al. 2023). Thus, larval density manipulation offers a standardized and efficient method for generating female size variation for male mate choice trials.

### 2.3 Collecting Experimental Males

We collected experimental males from the LH_M_ population using light CO_2_ anesthesia within 6 h of eclosion to ensure virginity. At the time of collection, males were randomly allocated to one of two treatment conditions. Males assigned to the “No Rivals” treatment were placed individually in vials containing a small amount of food medium. Males assigned to the “Rivals” treatment were placed in groups of five into separate vials containing a small amount of food medium. Holding males in groups of five for at least 24 h has been shown to induce plastic changes in male courtship and mating behavior compared to males held on their own (Bretman et al. 2009, 2010). All males were held under these conditions for 4 days prior to the onset of behavioral trials.

### 2.4 Data Collection Through a Course‐Based Undergraduate Research Experience (CURE) at Barnard College

Approximately half of the data presented in this work were collected by eight students enrolled in AP's Fall 2024 Laboratory in Animal Behavior course at Barnard College (biol bc2281), which was taught in a Course‐based Undergraduate Research Experience (CURE) format (Auchincloss et al. 2014; Bangera and Brownell 2014). As part of this CURE, each student collected and processed their own data (double‐checked by the teaching assistant for the course, HS, who also worked as an undergraduate researcher in AP's research lab) and each wrote a scientific paper about their findings. Seven students (RSH, TH, MJ, IMM, KLR, LES, and RZ) additionally chose to be involved in the preparation and editing of the manuscript after the course had completed. These CURE students collected behavioral data for one experimental block for each of Experiments 1 and 2 below. Each experiment was also replicated in 1–2 additional blocks by trained researchers in AP's research lab (including HS, AL, and AP) during the Spring 2025 semester.

### 2.5 Experiment 1: Testing the Impact of Exposure to Rivals on Precopulatory Male Mate Choice

We conducted male mate choice trials using previously established methods (Sinclair et al. 2021; Lev and Pischedda 2023; Freed et al. 2025) in which a single male was placed with one large female and one small female simultaneously to assess preferences in courtship and mating behavior. This experiment was conducted across three experimental blocks: one block was conducted by students in the CURE course and two blocks were conducted in AP's research lab. On the evening prior to each experimental block, observation vials were prepared by using a mouth aspirator to transfer one large virgin female and one small virgin female into a vial containing a small amount of food medium. These vials were held overnight in an incubator under standard conditions to allow females to acclimate.

When the incubator lights turned on the following morning, males from either the “Rivals” or “No Rivals” treatment groups were aspirated individually into the prepared vials containing both a large and a small female. The vials were labeled with treatment codes by a third party, such that the observers remained unaware of each male's treatment throughout the experiment. Labeled vials were then evenly distributed across either 8 or 4 observers, depending on the experimental block (eight student observers during the CURE course, four observers in AP's research lab). Each observer monitored 10 vials (five from each male treatment group). Immediately prior to the start of the observation period, foam plugs were gently pushed down into each vial to create an interaction space of approximately 1 cm in height. Observers then watched these vials continuously for 30 min at room temperature (~22°C) and recorded minute‐by‐minute data on male courtship and mating behavior. Specifically, courtship was scored for each minute in which the male either performed a courtship song (i.e., extended a wing and vibrated it while near a female) or attempted copulation, along with the target of the courtship (i.e., the large or small female). If a male courted both females during a single minute, both courtship events were recorded; however, multiple courtship episodes directed at the same female within a single minute were scored as a single event. If the male mated during the observation period, the identity of the mated female was recorded along with the minute mating began and the minute it ended. All matings that began within the 30‐min observation period were followed to completion, even if copulation continued past the initial observation window. Across all blocks, we collected data for 78 males from the Rivals treatment and 79 males from the No Rivals treatment.

### 2.6 Experiment 2: Testing the Impact of Exposure to Rivals on Postcopulatory Male Mate Choice and Female Offspring Production

In this study, we used mating duration as a proxy for postcopulatory cryptic male mate choice. Mating duration is primarily under male control in *D. melanogaster* (Jagadeeshan and Singh 2006; Friberg 2006; Bretman, Westmancoat, and Chapman 2013), and longer matings benefit males by delaying female remating and increasing male success at sperm competition (Anastasio et al. 2023; Martinez and Pischedda 2025). *D. melanogaster* males typically mate for longer with large females than with small females (Lefranc and Bundgaard 2000; Anastasio et al. 2023; Martinez and Pischedda 2025), but it is unclear whether being exposed to rivals affects the degree to which males extend matings with large females. We obtained some mating duration data in Experiment 1 above, but because most males mated with the large female (see Results), in Experiment 2 we paired each male with only one female of a given size to obtain more balanced sample sizes for matings with large and small females. This experiment was conducted across two experimental blocks: one conducted in the CURE course and one conducted in AP's research lab.

On the evening prior to the behavioral observations, we used a mouth aspirator to transfer one large virgin female or one small virgin female into individual food vials, creating four treatment groups for the following day: No Rivals/Large, No Rivals/Small, Rivals/Large, and Rivals/Small. The next morning, when the incubator lights came on, we aspirated males from either the Rivals or No Rivals treatment individually into the prepared vials. As for Experiment 1 above, all vials were coded and labeled by a third party so that observers remained unaware of the male treatment during data collection.

Vials were evenly distributed across observers depending on the experimental block. In the CURE course, eight student observers each monitored 15 vials, while in AP's research lab, four trained observers each monitored 30 vials. As in Experiment 1, foam plugs were pushed down into the vials to create an interaction space of approximately 1 cm in height. Observations were conducted at room temperature (~22°C) for 45 min. For all vials in which mating occurred, we recorded the minute mating began and the minute it ended. Any copulations initiated within the 45‐min observation period were watched to completion.

Female handling protocols at the end of the 45 min observation period differed between experimental blocks. In the CURE course block, all females were discarded following observations. In the block conducted in AP's research lab, we measured offspring production for females that had mated to Rivals versus No Rivals males by transferring mated females individually into fresh oviposition vials using a mouth aspirator. These vials contained ~7 mL of food medium that had been cut on the surface to stimulate oviposition. Females were held in these vials in the incubator for 27 h before being discarded. This oviposition period captured the majority of male‐mediated effects on short‐term female fecundity (Herndon and Wolfner 1995; Heifetz et al. 2000). Eleven days after female removal, when all offspring had eclosed, we counted the number of adult offspring in each vial.

Across the two experimental blocks, the mating duration dataset included a total of 52–62 males per male treatment/female size combination. Offspring production was only measured in the research lab block and included offspring from 25 to 28 females per treatment/size combination.

### 2.7 Data Processing and Analysis: Experiment 1

We obtained several metrics related to overall male courtship and mating behavior from our precopulatory male mate choice trials in Experiment 1. To determine whether exposure to rivals affected male courtship behavior, we measured courtship latency and overall courtship effort for each male. Courtship latency was calculated as the number of minutes the male took to initiate courtship (of any female) after the observation period began, while courtship effort was measured as the proportion of time the male spent courting either female. For males that mated during these trials, we calculated courtship effort as the number of minutes in which courtship was observed divided by the minute that mating began. For males that did not mate during the trials, the denominator was replaced with 30 (the total number of observation minutes).

To determine whether females responded differently to the courtship of “Rivals” or “No Rivals” males, we also compared mating success and the courtship threshold to mate between male treatments. For mating success, males that successfully mated (with either female) were coded as 1, while males that did not mate during the observation period were coded as 0. For males that mated during these trials, we used the courtship threshold to mate as a measure of the male's courtship effectiveness with the female he mated with. The courtship threshold to mate was calculated as the total number of minutes a male spent courting the specific female (large or small) that he ultimately mated with.

To compare the strength of precopulatory male preferences, we measured the proportion of courtship that was directed toward the large female for each male. To calculate this metric, we treated each minute in which the male courted as two possible courtship units, one for each female (large and small). If the male courted only the large female during a given minute, that minute was assigned a score of 2; if he courted both the large and small females during a given minute, it was assigned a score of 1; and if he only courted the small female during a given minute, it was assigned a score of 0. To calculate the proportion of courtship units directed toward the large female, we summed up the total score for a given male and divided it by the total number of courtship units (i.e., total number of minutes the male spent courting multiplied by two). This metric ranges from 0, indicating the male only courted the small female, to 1, indicating the male only courted the large female. A value of 0.5 indicates that the male courted the large and small females equally (i.e., had no preference). This measure of precopulatory male mate choice correlates perfectly with the “preference index” used to measure male mate choice in previous studies (Sinclair et al. 2021; Lev and Pischedda 2023) but allowed us to use a generalized linear mixed model (GLMM, see below) to compare male preferences between treatments (as in Freed et al. 2025). The proportion of courtship directed toward the large female was our main metric of precopulatory male mate choice, but as an additional metric, we calculated the total proportion of males from each treatment that mated with the large female (coded as a 1) versus the small female (coded as a 0). Finally, although Experiment 1 was intended to measure precopulatory male mate choice, more than 98% of males from both treatments mated in this experiment (see Results), so we also compared mating durations for Rivals and No Rivals males that mated with the large versus the small female.

All data analyses for Experiment 1 were conducted using mixed models in the *glmmTMB* package (version 1.1.11; Brooks et al. 2017) in R 4.5.1 (R Core Team 2025), with observer and experimental block as random effects. We treated block (*N* = 3) as a random effect in these analyses (Gomes 2022), but none of our conclusions changed if we ran the models with block as a fixed effect. We used a GLMM with a Poisson distribution to compare courtship latency between male treatments (Rivals or No Rivals) and a GLMM with a binomial distribution to compare courtship effort between treatments. For each model, we first confirmed that the data were not overdispersed using the *DHARMa* package (version 0.4.7; Hartig 2022). For the proportion of courtship directed toward the large female, we used a binomial GLMM (after confirming the data were not overdispersed) to compare courtship preferences between male treatments, and we used the *emmeans* package (version 1.11.1; Lenth 2023) to run 1‐sample Wald *z*‐tests to determine whether there was a significant male preference within each treatment (i.e., testing the null hypothesis that the mean proportion of courtship directed toward the large females was 0.5, indicating equal courtship of large and small females). We also used binomial GLMMs to compare mating success and the proportion of matings with large females between male treatments. For courtship threshold to mate and mating duration, we tested the full‐factorial effects of male treatment and the size of the female mated (large or small) using a Poisson GLMM and a linear mixed model, respectively (after confirming the data were not overdispersed). For all analyses, we obtained least squares (LS) means and 95% confidence intervals (CIs) using the *emmeans* package, which we also used to run pairwise tests for the courtship threshold to mate and mating duration analyses. We specified unadjusted *p*‐values for all tests run in *emmeans* (the 1‐sample Wald *z*‐tests for proportion of courtship directed toward the large female and the pairwise tests for courtship threshold to mate and mating duration) and then adjusted for multiple comparisons using the sequential Bonferroni correction (Holm 1979).

### 2.8 Data Processing and Analysis: Experiment 2

We used our mating data in Experiment 2 to test whether exposure to rivals affected male mating success and mating duration with large and small females. We compared mating success in *glmmTMB* using a binomial GLMM (with a successful mating = 1 and no mating = 0) that had observer added as a random effect and tested the full‐factorial fixed effects of male treatment, the size of the female mated, and experimental block. However, the inclusion of the 3‐way interaction term caused overparameterization issues with the model, so we removed it and reran the model with all possible 2‐way interactions. We then used a linear mixed model in *glmmTMB* with observer as a random effect to test the full‐factorial effects of male treatment, female size, and experimental block on mating duration. Finally, we measured offspring production by large and small females that had mated with Rivals and No Rivals males during the second block of Experiment 2. We compared the number of offspring produced using a negative binomial GLMM in *glmmTMB* that tested the full‐factorial fixed effects of male treatment and female body size.

For the mating success and mating duration analyses, we used the *car* package (version 3.1.3; Fox and Weisberg 2019) to obtain Analysis of Deviance tables. For all analyses above, we then used *emmeans* to obtain LS means, 95% CIs, and run pairwise tests. We specified unadjusted *p*‐values from these pairwise tests and then adjusted for multiple comparisons using the sequential Bonferroni correction (Holm 1979).

## 3 Results

### 3.1 Experiment 1: Does Exposure to Rivals Affect Male Courtship Behavior and Precopulatory Male Mate Choice?

**FIGURE 1:**
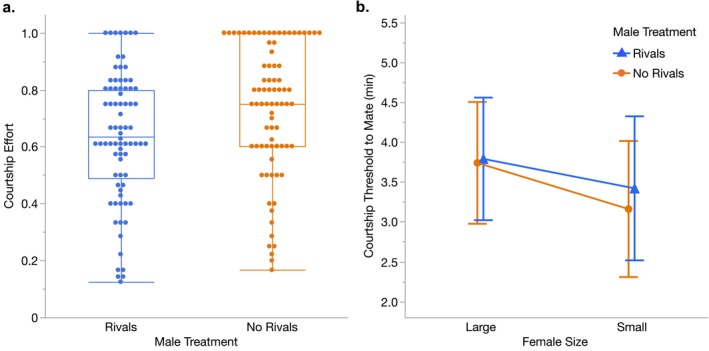
(a) Courtship effort and (b) courtship threshold to mate for males that had been held with or without rivals before the start of Experiment 1. (a) Courtship effort was measured as the proportion of time the male spent courting. Males that had been exposed to rivals had significantly lower courtship effort than males that had not been exposed to rivals (*p* < 0.0001, Table 1b). Each point represents an individual male (Rivals *N* = 78, No Rivals *N* = 79). (b) Courtship threshold to mate was measured as the total number of minutes the male courted a specific female (large or small) before mating with her. Shown are the least squares means ±95% confidence intervals (with Large Females: Rivals *N* = 53, No Rivals *N* = 53; with Small Females: Rivals *N* = 24, No Rivals *N* = 25). There was no significant effect of male treatment (*p* = 0.88) or female size (*p* = 0.43) on mean courtship threshold to mate (Table 1d).

We found that exposure to rivals affected both courtship latency and overall courtship effort. Compared to males that had been held on their own, males that had been exposed to rivals took longer to initiate courtship (LS mean courtship latency ±95% CI: Rivals = 1.9 ± 1.1 min, No Rivals = 0.9 ± 0.5 min; *p* < 0.0001, Table 1a) and had lower overall courtship effort (LS Mean proportion of time spent courting ±95% CI: Rivals = 0.63 ± 0.14, No Rivals = 0.77 ± 0.11; *p* < 0.0001, Table 1b, Figure 1a). Despite these differences in male courtship behavior, we found no evidence that females responded differently to males based on whether they had or had not been exposed to rivals. There was no difference between male treatments in overall mating success (LS mean proportion of successful matings ±95% CI: Rivals = 0.99 ± 0.04, No Rivals = 0.99 ± 0.04; *p* = 0.99, Table 1c) or in the courtship threshold to mate with large females (LS mean number of courtship minutes before mating ±95% CI: Rivals = 3.8 ± 0.8 min, No Rivals = 3.7 ± 0.8 min; *p* = 0.88, Table 1d) or with small females (Rivals = 3.4 ± 0.9 min, No Rivals = 3.2 ± 0.9 min; *p* = 0.63, Table 1d, Figure 1b).

**TABLE 1:** Effects of exposure to rivals on male courtship behavior, mating behavior, and precopulatory male mate choice in Experiment 1.

**(a) Courtship latency**			**(e) Proportion of courtship directed toward the large female**			
**Random effects**	**Variance**		**Random effects**	**Variance**		
Block	0.084		Block	0.013		
Observer	0.37		Observer	0.040		
**Fixed effect**	***z***	***p***	**Fixed effect**	***z***	***p***	
Male treatment	−5.60	< 0.0001	Male treatment	−1.40	0.16	
**(b) Courtship effort**			**Wald *z*‐tests of treatment means**	***z***	***p***	
**Random effects**	**Variance**		Rivals Males	5.4	< 0.0001	
Block	0.099		No Rivals Males	4.28	< 0.0001	
Observer	0.60		**(f) Size of female mated**			
**Fixed effect**	***z***	***p***	**Random effects**	**Variance**		
Male treatment	5.08	< 0.0001	Block	4.93E‐13		
**(c). Mating success**			Observer	2.47E‐09		
**Random effects**	**Variance**		**Fixed effect**	***z***	***p***	
Block	1.71E‐32		Male treatment	−0.12	0.91	
Observer	1.75E‐08		**(g) Mating duration**			
**Fixed effect**	***z***	***p***	**Random effects**	**Variance**		
Male treatment	0.009	0.99	Block	0.033		
**(d) Courtship threshold to mate**			Observer	0.21		
**Random effects**	**Variance**		**Fixed effects**	***z***	***p***	
Block	6.47E‐10		Male treatment	−4.20	< 0.0001	
Observer	6.54E‐02		Size of female mated	−5.60	< 0.0001	
**Fixed effects**	***z***	***p***	Male treatment × size of female tated	−0.30	0.77	
Male treatment	−0.15	0.88	**Pairwise tests**	***t***	**df**	***p***
Size of female mated	−0.80	0.43	Rivals large—No Rivals large	4.20	143	< 0.0001
Male treatment × size of female mated	−0.33	0.74	Rivals large—Rivals small	5.60	143	< 0.0001
**Pairwise tests**	***z***	***p***	No Rivals large—No Rivals small	5.32	143	< 0.0001
Rivals large—No Rivals large	0.15	0.88	Rivals small—No Rivals small	2.42	143	0.017
Rivals small—No Rivals small	0.49	0.63				

*Note:* Shown are the results from: (a) a Poisson GLMM testing for an effect of male treatment (Rivals or No Rivals) on courtship latency, (b) a binomial GLMM testing for an effect of male treatment on courtship effort, (c) a binomial GLMM testing for an effect of male treatment on mating success, (d) a Poisson GLMM testing the full‐factorial effects of male treatment and the size of the female mated (large or small) on the male's courtship threshold to mate, (e) a binomial GLMM testing the effect of male treatment on the proportion of courtship directed toward the large female, (f) a binomial GLMM testing for an effect of male treatment on the size of the female mated, and (g) a linear mixed model testing the full‐factorial effects of male treatment and the size of the female mated on mating duration. All models included experimental block and observer as random effects. Also shown for **e** are the results of 1‐sample Wald *z*‐tests for each treatment testing the null hypothesis that the mean proportion of courtship directed toward the large female was 0.5 (indicating no preference between large and small females). Also shown for **d** and **g** are the relevant pairwise comparisons. *P*‐values shown for the 1‐sample Wald tests (e) and the pairwise tests (d and g) are unadjusted for multiple comparisons and all *p*‐values that were < 0.05 remained significant after sequential Bonferroni corrections.

**FIGURE 2:**
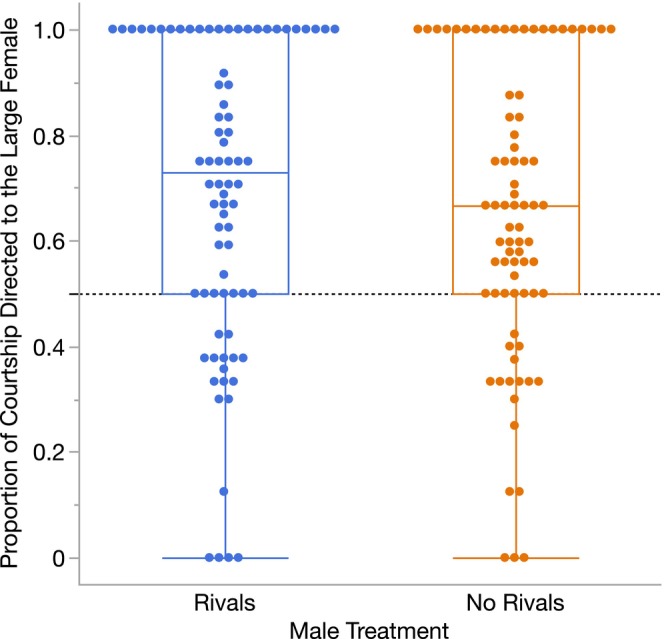
Precopulatory male mate choice for males that had been held with or without rivals before the start of Experiment 1. We measured precopulatory male preferences as the proportion of courtship directed toward the large female versus the small female. Each point represents an individual male (Rivals *N* = 78, No Rivals *N* = 79); the dashed horizontal line indicates the point at which males courted the large and small female equally (i.e., proportion = 0.5). Males that had been exposed to rivals and males that had not been exposed to rivals both had mean proportions that were significantly greater than 0.5 (both *p* < 0.0001), indicating that they spent more time courting the large female, on average. However, there was no significant difference between male treatments in the mean proportion of courtship directed toward the large female (*p* = 0.16, Table 1e).

When we tested for differences in the strength of precopulatory male preferences for large females, we found that males from both treatments had significant courtship preferences for large females (i.e., the proportion of courtship directed to the large female was > 0.5; both *p* < 0.0001, Table 1e, Figure 2), but there was no difference in the strength of this male preference between males that had and had not been exposed to rivals (LS mean proportion of courtship directed to the large female ±95% CI: Rivals = 0.66 ± 0.05, No Rivals = 0.63 ± 0.05; *p* = 0.16, Table 1e, Figure 2). We similarly found that Rivals and No Rivals males were equally likely to mate with the large female versus the small female (LS mean proportion of matings with the large female ±95% CI: Rivals = 0.69 ± 0.10, No Rivals = 0.68 ± 0.10; *p* = 0.91, Table 1f).

**FIGURE 3:**
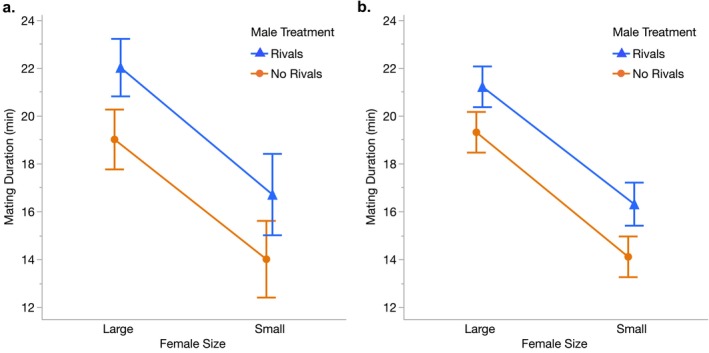
Mean mating durations with large and small females for males that had been held with or without rivals before the start of (a) Experiment 1 and (b) Experiment 2. Shown for both are the least squares means ±95% confidence intervals. Males held with rivals mated for significantly longer than males held without rivals in both experiments (**a:** *P* < 0.0001, Table 1g; **b:** *P* = 0.0094, Table 2b). Males from both treatments mated significantly longer with large females than with small females (**a:** *P* < 0.0001, Table 1g; **b:** P < 0.0001, Table 2b), but there were no significant interactions between male treatment and the size of the female mated (**a:** *P* = 0.77, Table 1g; **b:** *P* = 0.37, Table 2b), indicating that males from both treatments extended matings to the same degree with large females. Sample sizes were as follows: Experiment 1 (a): Rivals with large females *N* = 53, No Rivals with large females *N* = 53, Rivals with small females *N* = 24, No Rivals with small females *N* = 25. Experiment 2 (b): Rivals with large females *N* = 62, No Rivals with large females *N* = 60; Rivals with small females *N* = 52, No Rivals with small females *N* = 58.

Although the focus of this experiment was on precopulatory male behavior, the high mating success for males enabled us to also measure mating duration, our proxy for postcopulatory male mate choice. We first replicated past findings (Bretman et al. 2009; Bretman, Westmancoat, et al. 2011; Bretman, Westmancoat, and Chapman 2013; Wigby et al. 2009) showing that males that had been exposed to rivals mated for significantly longer than males held on their own (*p* < 0.0001; Table 1g, Figure 3a). This result was consistent when males mated with both large females (LS mean mating duration ±95% CI: Rivals = 22.0 ± 1.2 min, No Rivals = 19.0 ± 1.3 min; *p* < 0.0001; Table 1g, Figure 3a) and with small females (Rivals = 16.7 ± 1.7 min, No Rivals = 14.0 ± 1.6 min; *p* = 0.017; Table 1g, Figure 3a). Notably, males from both treatments mated significantly longer with large females than with small females (both *p* < 0.0001, Table 1g, Figure 3a), and there was no significant interaction between male treatment and female size (*p* = 0.77; Table 1g, Figure 3a), indicating that males from both treatments extended matings to the same degree with large females.

### 3.2 Experiment 2: Does Exposure to Rivals Affect Postcopulatory Male Mate Choice and Offspring Production?

Because most males mated with the large female in Experiment 1 above, in Experiment 2 we replicated the mating portion of that experiment but instead paired males with only a single large or single small female to balance the sample sizes. Consistent with our findings in Experiment 1, males exposed to rivals and males held on their own had comparable mating success with both large females (LS mean proportion of males that mated ±95% CI: Rivals = 0.98 ± 0.05, No Rivals = 0.97 ± 0.06; *p* = 0.69; Table 2a) and small females (Rivals = 0.91 ± 0.09, No Rivals = 0.92 ± 0.08; *p* = 0.87; Table 2a).

**TABLE 2:** Effects of exposure to rivals on male mating success, mating duration, and offspring production with large and small females in Experiment 2.

**(a) Mating success**			
**Random effect**	**Variance**		
Observer	0.29		
**Fixed effects**	***χ*** ^**2**^	**df**	***p***
Male treatment	0.86	1	0.35
Size of female mated	2.40	1	0.12
Male treatment × size of female mated	0.18	1	0.67
Block	2.14	1	0.14
Block × male treatment	2.19	1	0.14
Block × size of female mated	0.86	1	0.35
**Pairwise tests**	***z***	***p***	
Rivals large—No Rivals large	0.40	0.69	
Rivals small—No Rivals small	−0.16	0.87	
**(b) Mating duration**			
**Random effect**	**Variance**		
Observer	0.30		
**Fixed effects**	***χ*** ^**2**^	**df**	***p***
Male treatment	6.74	1	0.0094
Size of female mated	36.19	1	< 0.0001
Male treatment × size of female mated	0.79	1	0.37
Block	0.02	1	0.88
Block × male treatment	0.06	1	0.81
Block × size of female mated	0.05	1	0.83
Block × male treatment × size of female mated	0.53	1	0.47
**Pairwise tests**	***t***	**df**	***p***
Rivals large—No Rivals large	3.31	222	0.0011
Rivals large—Rivals small	8.46	222	< 0.0001
No rivals large—No Rivals small	9.37	222	< 0.0001
Rivals small—No Rivals small	3.83	222	0.0002
**(c) Offspring production**			
**Fixed effects**	***z***	***p***	
Male treatment	−1.00	0.32	
Size of female mated	−24.80	< 0.0001	
Male treatment × size of female mated	−0.07	0.94	
**Pairwise tests**	***z***	***p***	
Rivals large—No Rivals large	1.00	0.32	
Rivals large—Rivals small	24.80	< 0.0001	
No rivals large—No Rivals small	25.59	< 0.0001	
Rivals small—No Rivals small	0.50	0.62	

*Note:* Shown are the results for: (a) a binomial GLMM testing the full‐factorial effects of male treatment (Rivals or No Rivals) and the size of the female mated (large or small) on male mating success, (b) a linear mixed model testing the full‐factorial effects of male treatment and the size of the female mated on mating duration, and (c) a negative binomial GLMM testing the full‐factorial effects of male treatment and the size of the female mated on offspring production. The model in **a** includes observer as a random effect and all two‐way interactions between experimental block (as a fixed effect), male treatment, and female size. The model in **b** includes observer as a random effect and all full‐factorial interactions between experimental block (as a fixed effect), male treatment, and female size. Also shown for each model are the relevant pairwise comparisons; *p*‐values shown for these tests are unadjusted for multiple comparisons and all *p*‐values that were < 0.05 remained significant after sequential Bonferroni corrections.

When we looked at male mating duration, our proxy for postcopulatory male mate choice, we again found the same patterns as in Experiment 1. Males that had been exposed to rivals mated for significantly longer than males that had not been exposed to rivals (*p* = 0.0094; Table 2b, Figure 3b), and this finding held for matings with both large females (LS mean mating duration ±95% CI: Rivals = 21.2 ± 0.9 min, No Rivals = 19.3 ± 0.9 min; *p* = 0.0011, Table 2b, Figure 3b) and small females (Rivals = 16.3 ± 0.9 min, No Rivals = 14.1 ± 0.9 min; *p* = 0.0002, Table 2b, Figure 3b). Males from both treatments mated significantly longer with large females than with small females (both *p* < 0.0001, Table 2b, Figure 3b), and there was no interaction between male treatment and female size (*p* = 0.37, Table 2b, Figure 3b), so males from both treatments extended matings to the same degree with large females.

**FIGURE 4:**
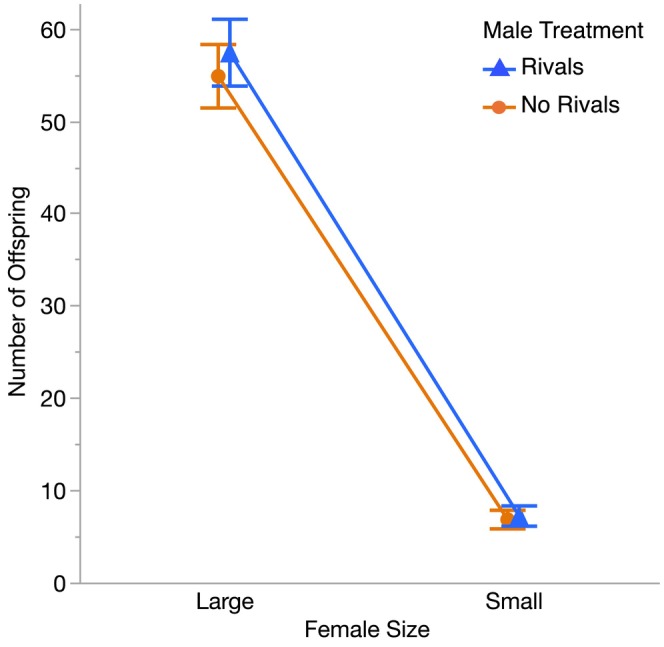
Mean offspring production for large and small females after mating with males that had been held with or without rivals before the start of Experiment 2. Shown are the least squares means ±95% confidence intervals (Large Females: Rivals *N* = 27, No Rivals *N* = 28; Small Females: Rivals *N* = 25, No Rivals *N* = 28). Large females produced significantly more offspring than small females (*p* < 0.0001), but there was no effect of male treatment (*p* = 0.32) and no male treatment by female body size interaction (*p* = 0.94, Table 2c) for offspring production.

Last, we tested whether the differences in mating duration between males exposed to rivals and males held on their own affected the number of offspring produced by the large and small females they mated with. While large females produced more offspring than small females, as expected (*p* < 0.0001, Table 2c, Figure 4), we found no differences in the number of offspring produced by large females or small females after mating with males from either treatment (LS mean number of offspring ±95% CI: Large females with Rivals males = 57.4 ± 3.6, Large females with No Rivals males = 54.8 ± 3.4, *p* = 0.32; Small females with Rivals males = 7.2 ± 1.1, Small females with No Rivals males = 6.8 ± 1.0, *p* = 0.62; Table 2c, Figure 4).

## 4 Discussion

Male–male competition is predicted to affect male mate choice in various ways, potentially strengthening male preferences when males benefit from investing more in fewer, higher‐quality matings, or weakening preferences when male choosiness leads to missed mating opportunities (Fawcett and Johnstone 2003; Servedio and Lande 2006; Servedio 2007; Bretman, Gage, and Chapman 2011; Edward and Chapman 2011; Fitzpatrick and Servedio 2018; Pollo et al. 2022). In *D. melanogaster* , exposure to rivals is known to alter male courtship and mating behaviors (Bretman et al. 2009, 2010; Bretman, Westmancoat, et al. 2011; Bretman, Westmancoat, and Chapman 2013; Wigby et al. 2009; Rouse and Bretman 2016; Dore et al. 2021; Fowler et al. 2022), but any effect of male competition on male mate choice had not yet been tested. Here, we investigated whether male competition influenced pre‐ and postcopulatory male mate choice for larger, higher‐fecundity females in *D. melanogaster* . While we replicated past work showing plasticity in male courtship and mating behaviors, males showed consistent pre‐ and postcopulatory preferences for large females regardless of the level of competition they had encountered. These findings suggest that male–male competition may have a limited impact on male mate choice in this system.

Exposure to rivals induced clear plasticity in male courtship behavior. Consistent with past work (Dore et al. 2021), males that had been held with rivals took significantly longer to initiate courtship than males that had not encountered rivals. This longer courtship latency likely contributed to the lower overall courtship effort for males that had been held with rivals (Figure 1a). While we might expect these males to start courting sooner and court more intensely in anticipation of heightened competition, their delayed courtship initiation and lower courtship effort may instead reflect more time spent evaluating females. Despite this possibility, we found no difference in the strength of precopulatory male preferences between males that had and had not encountered rivals, as males from both treatments directed significantly more courtship toward the large female than the small female (Figure 2).

In addition to this influence of male competition on courtship behavior, we also replicated past findings showing that exposure to rivals affected male mating duration (Bretman et al. 2009, 2010; Bretman, Westmancoat, et al. 2011; Bretman, Westmancoat, and Chapman 2013; Wigby et al. 2009; Rouse and Bretman 2016; Dore et al. 2020; Fowler et al. 2022). Specifically, males that had encountered rivals mated for significantly longer than males that had not encountered rivals, regardless of the size of the female they mated with (Figure 3). This plasticity in mating duration, which is largely under male control (Bretman, Westmancoat, and Chapman 2013), is consistent with an adaptive response to anticipated sperm competition, since longer matings are associated with delayed female remating and increased competitive fertilization success (Bretman et al. 2009; Martinez and Pischedda 2025). *D. melanogaster* males likely face higher rates of sperm competition with larger females, as these females remate more frequently (Anastasio et al. 2023; Martinez and Pischedda 2025). As a result, males exposed to rivals might be expected to extend matings with larger females to a greater degree than males not exposed to rivals. However, we found no evidence for this: males from both treatments showed the same relative difference in mating durations with large females versus small females (Figure 3). As with precopulatory male mate choice, male competition does not appear to affect the strength of male postcopulatory preferences for large females.

One possible explanation for this lack of an effect of male competition on male mate choice is that males from both treatments were equally effective at courting and mating with females. The predicted effects of male competition depend on the fitness benefits that males gain from their preferences (Fitzpatrick and Servedio 2018). In our study, males that had and had not been exposed to rivals had equal mating success and did not differ in their courtship threshold to mate (Figure 1b). Similarly, males from both treatments were equally likely to mate with the large female, and there were no differences in the number of offspring sired by males from the two treatments with either large or small females (Figure 4). While some studies have found that males exposed to rivals produced more offspring than males held on their own (Bretman et al. 2009, 2012; Bretman, Westmancoat, et al. 2011; Bretman, Westmancoat, Gage, and Chapman 2013; Hopkins et al. 2019), other work has failed to detect these benefits (Nandy et al. 2016; Dore et al. 2020; Fowler et al. 2022), consistent with our findings. Given that males from both treatments were similarly successful with preferred, larger females, we might not expect to see major differences in their pre‐ or postcopulatory preferences.

It is important to note, however, that males in our study were only exposed to rivals prior to encountering females, whereas the presence of competitors during courtship or mating might have a stronger effect on male mate choice. For example, males are predicted to be less choosy when there is more competition for preferred females and/or when rivals have a competitive advantage (Fawcett and Johnstone 2003; Servedio and Lande 2006; Servedio 2007; Edward and Chapman 2011), dynamics that are not captured in our study. However, experiments that varied the number of rivals present during courtship and mating did not find the plastic changes in mating duration seen when males were exposed to rivals prior to encountering females (Bretman et al. 2009), suggesting that this plasticity may take time to develop. As a result, it remains unclear whether the presence of rivals during male mate choice trials would affect male pre‐ or postcopulatory preferences. Additionally, our experiments only used virgin females, but males show similar plastic responses in mating duration with nonvirgin females (Bretman et al. 2009), so it is possible that exposure to rivals could affect the strength of pre‐ and/or postcopulatory preferences with nonvirgin females. Investigating how female mating status and the presence of rivals jointly affect male preferences could provide a more complete picture of the degree to which male–male competition shapes male mate choice in this system.

Together, our results add to a growing body of literature suggesting that pre‐ and postcopulatory male preferences for large, high‐fecundity females show limited plasticity in *D. melanogaster* (Sinclair et al. 2021; Anastasio et al. 2023; Lev and Pischedda 2023). Despite manipulating the social environment in a way that has well‐documented effects on male courtship and mating behaviors, we did not observe corresponding changes in male mate choice. These findings are consistent with past work showing that male mate choice for large females is similarly unaffected by a male's body size or mating history (Sinclair et al. 2021; Anastasio et al. 2023; Lev and Pischedda 2023). The stability in this preference suggests that selection may favor fixed over plastic strategies for male mate choice based on female body size.

## Author Contributions

**Harini Shankar:** investigation (lead), visualization (equal), writing – original draft (equal), writing – review and editing (equal). **Avigayil Lev:** investigation (supporting), writing – original draft (equal), writing – review and editing (equal). **Rachel S. Halpern:** investigation (supporting), writing – review and editing (supporting). **Thahiya Hassan:** investigation (supporting), writing – review and editing (supporting). **Mingxi Jiang:** investigation (supporting), writing – review and editing (supporting). **Isabella M. Martinez:** investigation (supporting), writing – review and editing (supporting). **Keane L. Robinson:** investigation (supporting), writing – review and editing (supporting). **Lauren E. Subramaniam:** investigation (supporting), writing – review and editing (supporting). **Rachel Zackai:** investigation (supporting), writing – review and editing (supporting). **Alison Pischedda:** conceptualization (lead), formal analysis (lead), funding acquisition (lead), investigation (supporting), methodology (lead), supervision (lead), visualization (equal), writing – original draft (equal), writing – review and editing (equal).

## Conflicts of Interest

The authors declare no conflicts of interest.

## Data Availability

All data and code associated with this paper are available at Dryad: https://doi.org/10.5061/dryad.v9s4mw77g.
